# Securing Cooperative Spectrum Sensing Against Collusive SSDF Attack using XOR Distance Analysis in Cognitive Radio Networks

**DOI:** 10.3390/s18020370

**Published:** 2018-01-27

**Authors:** Jingyu Feng, Man Zhang, Yun Xiao, Hongzhou Yue

**Affiliations:** 1 Shaanxi Key Laboratory of Information Communication Network and Security, Xi’an University of Posts & Telecommunications, Xi’an 710121, China; fengjy@xupt.edu.cn (J.F.); ZhangMan3418@163.com (M.Z.); 2 School of Information Science and Technology, Northwest University, Xi’an 710127, China; 3 Information Security Research Center of State Key Laboratory of Integrated Services Networks, Xidian University, Xi’an 710071, China; yuehz@nipc.org.cn

**Keywords:** cooperative spectrum sensing, cognitive radio, trust, collusive attack, network security

## Abstract

Cooperative spectrum sensing (CSS) is considered as a powerful approach to improve the utilization of scarce spectrum resources. However, if CSS assumes that all secondary users (SU) are honest, it may offer opportunities for attackers to conduct a spectrum sensing data falsification (SSDF) attack. To suppress such a threat, recent efforts have been made to develop trust mechanisms. Currently, some attackers can collude with each other to form a collusive clique, and thus not only increase the power of SSDF attack but also avoid the detection of a trust mechanism. Noting the duality of sensing data, we propose a defense scheme called XDA from the perspective of XOR distance analysis to suppress a collusive SSDF attack. In the XDA scheme, the XOR distance calculation in line with the type of “0” and “1” historical sensing data is used to measure the similarity between any two SUs. Noting that collusive SSDF attackers hold high trust value and the minimum XOR distance, the algorithm to detect collusive SSDF attackers is designed. Meanwhile, the XDA scheme can perfect the trust mechanism to correct collusive SSDF attackers’ trust value. Simulation results show that the XDA scheme can enhance the accuracy of trust evaluation, and thus successfully reduce the power of collusive SSDF attack against CSS.

## 1. Introduction

In a recent study made by the Federal Communications Commission, it was found that most of the licensed radio frequency spectrum is not efficiently utilized by the primary users [1]. In order to improve spectrum utilization, it has been suggested that opportunistic access to the spectrum should be given to secondary users [2]. Cognitive Radio is an emerging technology that would allow an secondary user (SU) to sense and use any available valid spectrum from primary users (PU) at a given time.

To avoid the case of deep shadowing and multipath fading, cooperative spectrum sensing (CSS) [3,4,5] has been considered as a viable method to enhance the detection performance by exploiting spatial diversity via the observations of spatially located SUs. However, CSS is often established randomly among SUs that are unrelated and unknown with each other [6], and thus offering opportunities for attackers to manipulate sensing data by launching SSDF attack [7]. Such SSDF attack pattern can be launched by two ways: individual or collusive. Compared with collusive attack, individual attack is less harmful and can be suppressed. In the collusive pattern, the attackers who collude with each other to form a collusive clique can increase the power of SSDF attack and fake the sensing data intentionally. If there are the adequate attackers, a collusive clique can lead to a wrong spectrum sensing decision.

To suppress SSDF attack, various trust mechanism studies have been presented [8,9,10,11]. They evaluate whether an SU is trusted or not by his historical sensing behaviors and give the low weights to less trusted SUs or even delete their sensing data when making a final decision. Nevertheless, collusive SSDF attackers can improve their trust value with the help of each other, except for increasing the attack power. Therefore, they may bypass the detection of the trust mechanism.

In this paper, we analyze the characteristics of collusive SSDF attack and present a defense scheme called XDA to suppress such an attack. The main contributions of this paper are as follow:

- Analyze the three types of threats of collusive SSDF attackers in detail. The first one is profit-driven, in which collusive SSDF attackers conspire with each other to falsify the sensing data inspired by some profits such as monopolizing vacant PU spectrums. The second is manipulating the trust mechanism, in which collusive SSDF attackers can improve their trust value quickly. The third is disturbing data fusion, in which collusive SSDF attackers can submit their false sensing data to disturb the data fusion of FC successfully with high trust value.
- Noting the duality of sensing data submitted by collusive SSDF attackers, the XOR distance calculation in line with the type of “0” and “1” historical sensing data is introduced. Based on the fact that collusive SSDF attackers hold the the lower XOR distance and high trust value simultaneously, a lightweight algorithm to detect collusive SSDF attackers is designed.
- Enhance the accuracy of trust evaluation. By reducing the increase of “the number of honest sensing data” with XDA, collusive SSDF attackers will not get a high trust value again. As a result, they can be detected by a trust mechanism.

The remainder of this paper is organised as follows: In Section 2, preliminaries related on CSS and trust mechanism are described. We analyze collusive SSDF attack and constructs the XDA scheme to suppress it in Section 3. Simulation analysis of the XDA scheme is performed in Section 4. Finally, we conclude this paper in Section 5.

## 2. Preliminaries

### 2.1. Cooperative Spectrum Sensing

**Figure 1:**
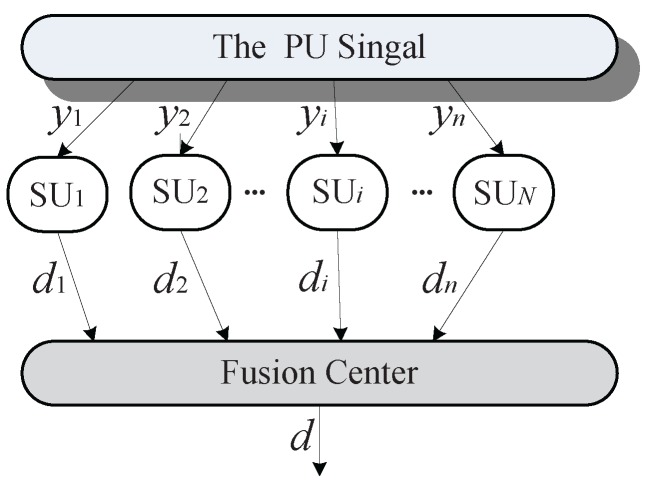
Modeling CSS as a parallel fusion network.

The CSS process can be modeled as a parallel fusion network [10]. As shown in Figure 1, a central identity called fusion center (FC) controls the process of CSS: individual sensing, data reporting and data fusion [12]. First, each SU exploits the energy detection to sense the signal of a PU via the sensing channel which is the selected licensed frequency band where a physical point-to-point link between the PU transmitter and each SU for observing the primary spectrum. Second, all SUs report their sensing data to FC via the reporting channel, which is a control channel with a physical point-to-point link between each SU and FC for sending individual sensing information. Finally, FC fuses the received individual sensing data as a single decision value to determine the presence of PU. Such a final decision can be made according to three typical data fusion rules: the “AND”, “OR” and “Majority” rule [13].

Typically, the energy detection method is used to detect a PU signal, and thus the individual sensing for energy detection method can be depicted as a binary hypothesis problem [14]:

(1) $$y\left(t\right)=\left\{\begin{array}{ll} n\left(t\right), & H_{0}\\ h\left(t\right)\cdot s\left(t\right)+n\left(t\right), & H_{1} \end{array}\right.$$

where y(t) is the sensed signal at each SU, s(t) is the transferred PU signal, h(t) is the channel gain of the sensing channel, n(t) is the zero-mean additive white Gaussian noise, and *t* is the sample index. $H_{0}$ and $H_{1}$ represent the hypothesis of the inexistence and the existence of the PU signal, respectively. If y(t) is larger than the decision threshold of energy detection, the existence of PU can be signal declared. Otherwise, no PU signal is detected.

After the individual sensing, the sensing data of each SU is determined. $d_{i}$ represents the sensing data of $SU_{i}$, which is generally described as a binary variable:

(2) $$d_{i}=\left\{\begin{array}{cc} 0, & H_{0}\\ 1, & H_{1} \end{array}\right.$$

where “0” and “1” indicate the hypothesis of the inexistence and existence of the PU spectrum status, respectively. Correspondingly, the final decision of FC is also binary via the data fusion. In CSS, the common methods of the data fusion are the “AND”, “OR” and “Majority” rule. In the “AND” rule, FC makes *d* = 1 when all $d_{i}$ = 1. The “OR” rule refers to *d* = 1 when one $d_{i}$ = 1. The “Majority” rule requires at least half of SUs to report “1”. The “OR” rule works the best if the number of SUs is large, whereas the “AND” rule performs well if the number of cooperating SUs is small, and the “Majority” rule can be derived from the *k* out of *N* rule under the case k≥N/2 [3].   

### 2.2. Trust Mechanism

Trust mechanism has been widely used in many application scenarios, including e-commerce [15], P2P network [16], ad hoc network [17], online social communities [18], etc.

Currently, the trust mechanism also plays a significant role in the CSS area. Representative trust mechanism schemes in CSS are as follows. In [8], the authors propose a trust-aware hybrid spectrum sensing scheme with the Beta reputation. However, they make the base station provide its sensing result for the trust weighted sensing result aggregation at each CSS action, which will cause a heavy overload to the base station if it also needs to detect the PU singal at each CSS action. Zeng et al. propose a secure CSS scheme with the assistance of trusted SUs to mitigate SSDF attack in [9], but a great deal of probability analysis used to identify attackers can lead to the increase of the computation complexity. In [10], the authors use a reputation based method called weighted sequential probability ratio test, but this method needs the location of PU and SUs to obtain some required prior probabilities and requires large number of samples and in worst case may lead to a deadlock situation with an endless sensing sampling. In [11], the authors propose a multi-factor trust management scheme by involving multiple decision factors, including the history-based trust factor, active factor, incentive factor and consistency factor, but the evaluation of these factors has caused more mathematical computation.

To avoid the heavy overload and suppress collusive SSDF attack simultaneously, we design a lightweight detection algorithm of collusive SSDF attackers in the basis of reducing the mathematical complexity of trust mechanism. Noting that the sensing data from SUs can be viewed as a binary variable (“0” or “1”), it is easy for them to produce two types of sensing results: honest or false. Based on the binary variable, the design idea of XOR distance analysis can be introduced to suppress collusive SSDF attack. Obviously, the fast XOR operation built on the “0” and “1” sensing data can make the detection algorithm lightweight, in which the SUs with the lower XOR distance and high trust value will be detected as collusive SSDF attackers.

Based on the binary variables, we can also abstract a simple trust mechanism scheme called Baseline, in which the trust value of each SU can be initialized by two indexes: the number of honest sensing (*hon*) and the number of false sensing (*fal*). After detecting collusive SSDF attackers, the Baseline can be perfected by preventing the increase of *hon*.

Generally, the beta function is one of the most popular designs using binary input (i.e., positive or negative) to evaluate trust value [8]. It counts the number of positive and negative behaviors a user has performed, and then calculates the trust value with beta probability density function denoted by Beta(α,β) [19].

(3) $$Beta\left(\alpha ,\beta \right)=\frac{\Gamma (\alpha +\beta )}{\Gamma (\alpha )\Gamma (\beta )}\theta^{\alpha -1}{\left(1-\theta \right)}^{\beta -1}$$

where θ is the probability of behaviors, 0≤θ≤1, α>0, β>0.

Take the *i*-th SU (SU${}_{i}$) as an example, $hon_{i}$ and $fal_{i}$ denote the number of honest sensing (positive) and false sensing (negative) conducted by SU${}_{i}$. In the Baseline, the trust value of SU${}_{i}$ can be calculated as

(4) $$T_{i}=Beta\left(hon_{i}+1,fal_{i}+1\right)$$

Consider the condition Γ(n)=(n−1)! when *n* is an integer [20]. Thus, the expectation value of the beta function is E[Beta(α,β)]=α/(α+β). In this case, $T_{i}$ can be further calculated as

(5) $$T_{i}=\frac{1+hon_{i}}{2+hon_{i}+fal_{i}}$$

Let δ denote the threshold of trust value. For $T_{i}\geq \delta$, $SU_{i}$ will be identified as an attacker, and vice versa. In order to guarantee the performance of CSS, δ should satisfy two requirements: (1) δ should be a rational value between 0 and 1 as $T_{i}\in \left[0,1\right]$; (2) the value of δ can be adjusted to suppress malicious responses generated by attackers who submit false sensing data.

Obviously, δ cannot be set as a low value in [0,1]. If this was done, then the attackers with high trust value would get more opportunities to submit false sensing data, resulting in the most malicious responses. In addition, δ cannot be set as the maximum of trust value since sometimes honest SUs may submit false sensing data at a lower probability due to the case of deep shadowing and multipath fading. To find the rational value of δ, the simulation method is a good choice. In this paper, we perform the simulation of suppressing malicious responses in Section 4, and find the rational value of δ is 0.8.

## 3. Collusive SSDF Attack and Defense Scheme

In this section, we first describe collusive SSDF attack, and then propose a defense scheme by using XOR clustering analysis called XDA to suppress collusive SSDF attack.

### 3.1. Collusive SSDF Attack Overview

Because the individual sensing report is usually regarded as a binary variable, it is very easy for attackers to launch an SSDF attack by submitting false individual sensing data, resulting in a wrong final decision of FC.

At first, attackers launch an SSDF attack individually and respectively. The power of this individual SSDF attack is finite and can be suppressed by a current trust mechanism easily, such as [8,9,10,11]. To avoid the detection of trust mechanism, some attackers attempt to collude with each other and submit false sensing data collusively at the same time. This attack pattern can be called a collusive SSDF attack. Generally, the collusive attack pattern can increase the power of attackers, which has undergone three stages.

In the first stage, an attacker can acquire multiple IDs to falsify data through the sybil attack [21]. There are many methods to defend against the Sybil attacks [22,23]. In particular, if each IP is restricted to acquire an ID, this attack pattern can be addressed easily. In the second stage, an attacker can control multiple computers by embedding trojan viruses. This attack pattern can be suppressed by using a good antivirus software. In the third stage, multiple attackers collaborate together to falsify the data. In this attack pattern, each attacker only has an ID. Currently, this attack pattern is used as a popular collusive attack, especially in CSS to falsify sensing data.

By further analyzing the characteristics of CSS and the collusive attack demand, we have found that three types of threats can be achieved by collusive SSDF attackers, namely: profit-driven, manipulate trust mechanism and disturb data fusion.

- **Profit-driven**: Inspired by some profits, collusive SSDF attackers can conspire with each other to form a collusive clique to falsify the sensing data intentionally. For example, they can monopolize vacant PU spectrums in the CSS environment. They submit false sensing data together to show the spectrum of a PU is in use, although it is idle. In this case, other SUs would recognize that the licensed spectrum is present and would not use the spectrum. Thus, collusive an SSDF attackers clique can gain exclusive access to the target licensed spectrum.
- **Manipulate trust mechanism**: By collusion, collusive SSDF attackers can improve their trust value quickly. For example, one of the collusive SSDF attackers who knows the actual status of a PU spectrum would tell this PU spectrum status to his conspirers in advance, and then sends a query message to the FC. Their trust values can be improved quickly if their sensing data are as consistent as the PU spectrum status.
- **Disturb data fusion**: As we know, the most primal attackers need to disturb some systems. With high trust value, collusive SSDF attackers can bypass the detection of the trust mechanism, and thus submit their false sensing data to indicate that the spectrum band of a PU spectrum is idle. As a result, they can disturb the data fusion of FC successfully, and ultimately deceive honest SUs to interfere PUs. In fact, SUs must never interfere with PUs in CSS [2]. If cause any interference to PUs, the availability of CSS will be questioned.

### 3.2. Design of XDA Scheme

To design the defense scheme of collusive SSDF attack, we analyze its attack threats, and thus find three kinds of general features as follows.

- **Duality**: SUs often submit the type of “0” and “1” sensing data to represent the hypothesis of the inexistence and the existenc of PU spectrum status. Thus, the sensing behaviors of SUs in the CSS environment can be abstracted as the duality due to the type of “0” and “1” sensing data.
- **Action together**: Collusive SSDF attackers always submit false sensing data together no matter which threats they would launch. They can fake “1” data together to monopolize vacant PU spectrums, or fake “0” data together to disturb the data fusion.
- **High trust value**: Collusive SSDF attackers often have high trust value. With the help of each other, they can improve their trust value by manipulating the trust mechanism.

Considering the “Duality” and “Action together” of general features, we introduce the design idea of XOR distance analysis to construct the defense scheme called XDA to suppress collusive SSDF attack. Meanwhile, the “Action together” of the general feature can make collusive SSDF attackers behave like the lower XOR distance among themselves. Based on this, we analyze the “High trust value” of a general feature to design the algorithm of detecting collusive SSDF attackers.

**Figure 2:**
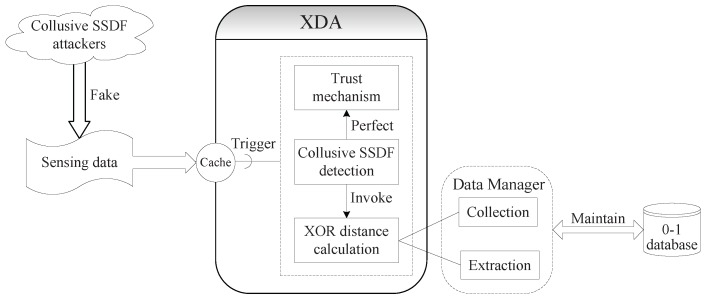
Architectural view of the XDA scheme.

As shown in Figure 2, the XDA scheme is conducted in three successive stages: XOR distance calculation, collusive SSDF detection and perfect trust mechanism. In the first stage, we design the XOR distance calculation between any two SUs in line with the type of “0” and “1” sensing data. In the second stage, an algorithm is designed to detect collusive SSDF attackers. In the third stage, we can perfect trust mechanism by correcting collusive SSDF attackers’ trust value after detecting them at each CSS action.   

#### 3.2.1. XOR Distance Calculation

It can be seen in Figure 2 that a data manager is responsible for performing two main tasks in this stage. It first collects the sensing data during each CSS action, in which FC is required to store the type of “0” and “1” historical sensing data to a small database called the 0-1 database, rather than discarding them again. The 0-1 database is designed as an extensible database, whose size corresponds to the sensing times of CSS actions. After each CSS action, FC should add a row in the 0-1 database to record the sensing data reported by cooperating SUs. When the current CSS action is numbered as the *k*-th sensing time, the size of the 0-1 database is *k*. The description of the 0-1 database is shown in Table 1.

Take $SU_{i}$ as an example, $SU_{i}{\left(d_{i}\right)}_{k}$ is recorded as $SU_{i}{\left(1\right)}_{k}$ when $SU_{i}$ reported “1” at the *k*-th sensing time, $SU_{i}{\left(d_{i}\right)}_{k}\to SU_{i}{\left(0\right)}_{k}$ when reported “0” and $SU_{i}{\left(d_{i}\right)}_{k}\to SU_{i}{\left(-\right)}_{k}$ when reported nothing.

Generally, distance metrics play a very important role in order to measure the similarity among the data sets [24]. For the convenience of calculating the distance between any two SUs, the second task of the data manager is to extract each cooperating SU’s sensing data from the 0-1 database as a vector in the current CSS action. For $SU_{i}$, his sensing vector can be represented as $D_{i}=\left[SU_{i}{\left(d_{i}\right)}_{1},SU_{i}{\left(d_{i}\right)}_{2},\cdots ,SU_{i}{\left(d_{i}\right)}_{k}\right]$. If $SU_{i}{\left(d_{i}\right)}_{1}\to SU_{i}{\left(1\right)}_{1}$, $SU_{i}{\left(d_{i}\right)}_{2}\to SU_{i}{\left(-\right)}_{2}$, $SU_{i}{\left(d_{i}\right)}_{k}\to SU_{i}{\left(0\right)}_{k}$, $D_{i}$ can be definitely described as [ 1, -,⋯, 0].

Obviously, the redundant data such as $SU_{i}{\left(-\right)}_{2}$ or $SU_{j}{\left(-\right)}_{5}$ are useless to calculate the distance between $SU_{i}$ and $SU_{j}$. In this case, we can perfrom Procedure 1 to eliminate the redundant data for the two SUs.

**Procedure 1** Eliminate redundancy
**Input:** $D_{i}$, $D_{j}$**Output:** $\tilde{D_{i}}$, $\tilde{D_{j}}$1:Initialize $\tilde{D_{i}}=\tilde{D_{j}}=\emptyset$2:**for** k=1,k≤n,k++ **do**3: **if** $\left(SU_{i}{\left(d_{i}\right)}_{k}\to SU_{i}{\left(-\right)}_{k}\right)||\left(SU_{j}{\left(d_{j}\right)}_{k}\to SU_{j}{\left(-\right)}_{k}\right)$ **then**
4:  $SU_{i}{\left(d_{i}\right)}_{k}$ and $SU_{j}{\left(d_{j}\right)}_{k}$ are deleted simultaneously;5: **else**6:  $\tilde{D_{i}}=\left\{SU_{i}{\left(d_{i}\right)}_{k}\right\}\cap \tilde{D_{i}}$
7:  $\tilde{D_{j}}=\left\{SU_{j}{\left(d_{j}\right)}_{k}\right\}\cap \tilde{D_{j}}$
8: **end if**9:**end for**


For all the cooperating SUs at the current sensing time, their sensing vector without the redundant data can compose a matrix ${\tilde{D}}_{h\times h}$.

$${\tilde{D}}_{h\times h}=\left(\begin{array}{ccc} d_{11} & \cdots & d_{1h}\\ \vdots & \ddots & \vdots \\ d_{h1} & \cdots & d_{hh} \end{array}\right)$$

For $SU_{i}$ and $SU_{j}$, the XOR operation between $\tilde{D_{i}}$ and $\tilde{D_{j}}$ can be described as

(6) $$\tilde{D_{ij}}=\tilde{D_{i}}⊕\tilde{D_{j}}$$

Then, the XOR distance between $SU_{i}$ and $SU_{j}$ can be calculated as

(7) $$xd_{ij}=\sum_{d_{ij}\left(k\right)\in \tilde{D_{ij}},k=1}^{|\tilde{D_{ij}}|}k*d_{ij}\left(k\right)$$

where $d_{ij}\left(k\right)$ is the *k*-th element of $\tilde{D_{ij}}$ and $|\tilde{D_{ij}}|$ is the number of $\tilde{D_{ij}}$.

For $SU_{i}$, the XOR distance related to the cooperating SUs at the current CSS action can compose the XOR distance vector ${\mathrm{XD}}_{i}=\left\{xd_{i1},\cdots ,xd_{ij},\cdots ,xd_{ih}\right\}$, in which *h* is the number of the cooperating SUs.

It is necessary to normalize the XOR distance in ${\mathrm{XD}}_{i}$. Otherwise, some SUs may be assigned arbitrarily high XOR distance with $SU_{i}$, and another SUs may have an arbitrarily low XOR distance, which introduces a difficulty in comparing them. To ensure that all XOR distance of $SU_{i}$ lie in [0, 1], $xd_{ij}$ can be normalized as

(8) $${\bar{xd}}_{ij}=\frac{xd_{ij}}{max({\mathrm{XD}}_{i})},xd_{ij}\in {\mathrm{XD}}_{i}$$

For all cooperating SUs, their normalized XOR distance can form a matrix ${\bar{XD}}_{h\times h}$.

$${\bar{XD}}_{h\times h}=\left(\begin{array}{ccc} {\bar{xd}}_{11} & \cdots & {\bar{xd}}_{1h}\\ \vdots & \ddots & \vdots \\ {\bar{xd}}_{h1} & \cdots & {\bar{xd}}_{hh} \end{array}\right)$$

#### 3.2.2. Collusive SSDF Detection

We know that collusive SSDF attackers often fake sensing data together, so there may be a low XOR distance between them. With the help of each other, collusive SSDF attackers can get high trust value when $T_{j}>\delta$, but the lower XOR distance will make them exposed. So, we can detect the SUs who hold high trust value and the minimum from all normalized $XD_{i}$ (i∈1,2,⋯,h) as the collusive SSDF attackers set ($\Upsilon_{1}$). In contrast, honest SUs also have high trust value, but there is a higher XOR distance between them. So, we can identify the SUs who hold high trust value and the maximum from from all normalized $XD_{i}$ as the honest SUs set ($\Upsilon_{2}$). Assuming that $\Gamma =\{T_{1},\cdots ,T_{j},\cdots ,T_{h}\}$ is the trust value set of cooperating SUs, an algorithm is designed in Procedure 2 to detect collusive SSDF attackers.

**Procedure 2** Detect collusive SSDF attackers
**Input:** Γ and ${\bar{XD}}_{h\times h}$**Output:** $\Upsilon_{1}$ and $\Upsilon_{2}$1:Initialize $\Upsilon_{1}=\Upsilon_{2}=\emptyset$2:**for** i=1,i≤h,i++ **do**
3: Initialize $\Omega_{i}=\emptyset$4: **for** j=1,j≤h,j++ **do**
5:  **if** $T_{j}>\delta$ **then**
6:   $\Omega_{i}=\left\{{\bar{xd}}_{ij}\right\}\cup \Omega_{i}$
7:  **end if**
8: **end for**
9: $\Upsilon_{1}=\left\{argmin\left(\Omega_{i}\right)\right\}\cup \Upsilon_{1}$10: $\Upsilon_{2}=\left\{argmax\left(\Omega_{i}\right)\right\}\cup \Upsilon_{2}$11:**end for**


To ensure the reliability of the data fusion, the sensing data of collusive SSDF attackers should be deleted. Of course, the sensing data of the SUs whose trust values are less than δ should also be deleted. Only the sensing data of honest SUs can be adopted in the data fusion.

#### 3.2.3. Perfect Trust Mechanism

When collusive SSDF attackers are detected, typical issues in perfecting the trust mechanism focus on (1) preventing the increase of their *hon* data in the current CSS action; (2) reducing their *hon* data until $T_{i}<\delta$; and (3) deleting their sensing data in the process of data fusion, which can be performed by Procedure 3. For the former two issues, collusive SSDF attackers will not get high trust value, thus enhancing the accuracy of trust evaluation. For the last one, collusive SSDF attackers will find it hard to manipulate the final decision of FC.

Let *H*, *F* is the set of current SUs’ *hon* data and *fal* data, respectively. For $SU_{i}\in \Theta$ (the set of cooperating SUs), $hon_{i}\in H$ and $fal_{i}\in F$. As mentioned in Figure 1, $d_{i}$ is the individual sensing data from $SU_{i}$ and *d* is the final decision from FC.

**Procedure 3** Perfect trust mechanism
**Input:** Θ, Γ, $\Lambda_{1}$ and $\Lambda_{2}$**Output:** *H*, *F*1:**for** each $SU_{i}\in \Theta$ **do**2: **if** $SU_{i}\in \Lambda_{1}$ **then**
3:  $hon_{i}=hon_{i}+0$ and his sensing data are deleted4:  **if** $T_{i}\geq \delta$ **then**
5:   **repeat** $hon_{i}--$6:   **until** $T_{i}<\delta$7:  **end if**
8: **else**
9:  **if** $d_{i}==d$ **then**
10:   $hon_{i}=hon_{i}+1$11:  **else**
12:   $fal_{i}=fal_{i}+1$13:  **end if**
14: **end if**
15:**end for**



## 4. Simulation Results and Discussion

We perform simulations to validate the performance of the XDA scheme and discuss the simulation results. The general simulation setup is shown in Table 2.

The simulations are performed by cycle-based fashion. At each cycle, SUs are selected randomly to execute CSS actions with each other. After several cycles, a trusted network topology is gradually generated by trust mechanism. FC then utilizes it to execute the following CSS actions, and update the trust value on the corresponding SUs. In addition, the case of deep shadowing and multipath fading can cause false detection for honest SUs. Therefore, sometimes, honest SUs may submit false sensing data at a lower probability in CSS. Without loss of generality, the behavior pattern for honest SUs in the simulations is modeled to submit false sensing data at the probability of 0.2.

**Figure 3:**
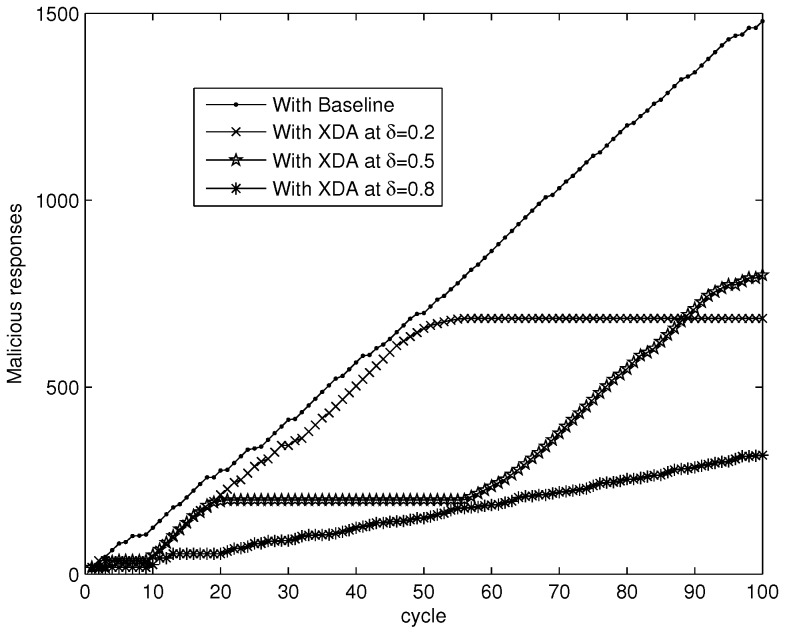
Suppressing malicious responses.

To perform the simulations better, it is necessary to select a rational value of δ. As $T_{i}\in \left[0,1\right]$, δ can be considered from the three types of optional states [low, medium, high]. Then, we can perform the simulation of suppressing malicious responses to validate the effectiveness of the XDA scheme under the three types of optional states of δ. In this simulation, 0.3, 0.5 and 0.8 denotes the low, medium and high state of δ respectively. As shown in Figure 3, the performance of the XDA scheme at δ=0.8 is the best. Therefore, the rational value of δ should be selected as 0.8 in the simulations. We can also see that the XDA scheme is better than Baseline at suppressing malicious responses, even though δ is selected as 0.8 for Baseline.

**Figure 4:**
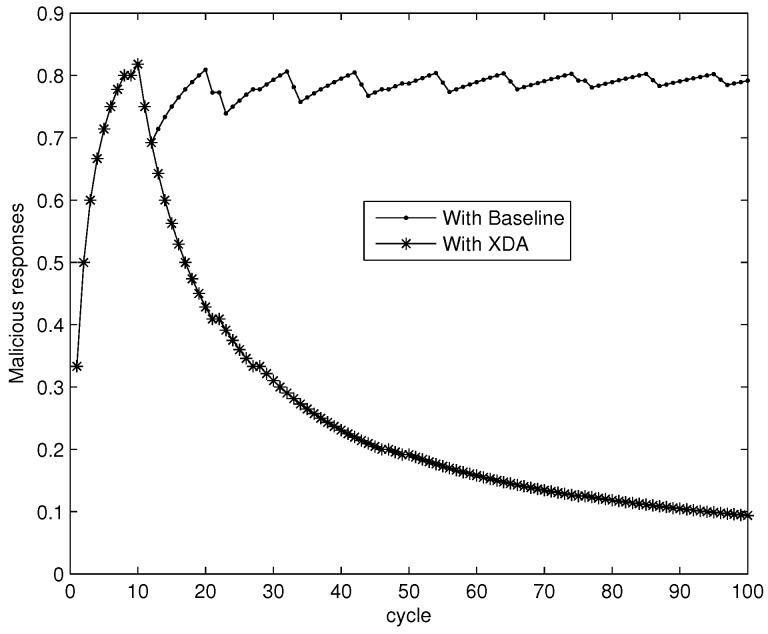
Variation of a collusive SSDF attacker’s trust value.

To suppress a collusive SSDF attack, an important measure is to suppress the increase of collusive SSDF attackers’ trust value. Therefore, we choose a collusive SSDF attacker randomly to observe the variation of his trust value with Baseline and XDA. Figure 4 shows that collusive SSDF strategies make the attacker’s trust value fluctuate along with the various cycles. The trust value usually outweighs δ in Baseline. Fortunately, the trust value can be reduced by the XDA scheme after 10 cycles. This is because the XDA scheme can delete *hon* data of collusive SSDF attackers and reduce this data in the trust mechanism. In addition, collusive SSDF attackers will deviate from the real trust value by forming high-trust attackers, and thus cause some network trust errors (*nte*). Higher errors indicate lower accuracy in calculating the trust value. The *nte* can be specified by:

(9) $$nte=\frac{1}{N}\sum_{i=1}^{N}\sqrt{\frac{1}{T_{i}^{\prime }}{\left(T_{i}^{\prime }-T_{i}\right)}^{2}}$$

where $T_{i}^{\prime }$ and $T_{i}$ are the real and measured trust value of $SU_{i}$, respectively.

Generally, the power of attackers is limited when the percentage of attackers decrease below 10%, but the network may become unavailable when the percentage of attackers decreases more than 50%. It can be found in Figure 4 that the network trust errors with Baseline is below 0.08 when the percentage of collusive attackers decreases below 10%, but the number of network trust errors is greater than 0.42 when the percentage of collusive attackers decreases by more than 50%. Thus, we vary the percentage of collusive attackers from 10% to 50% in the following simulations.

**Figure 5:**
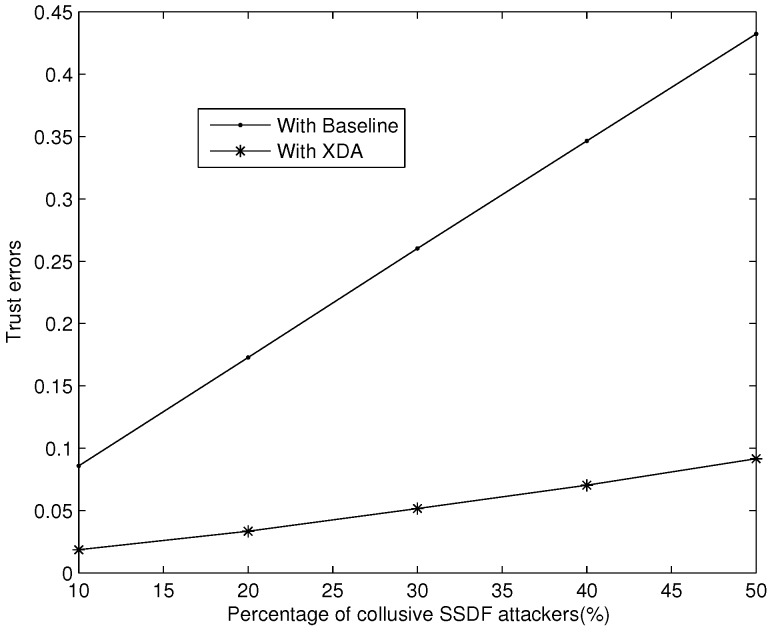
*nte* with the guard of XDA.

In the simulation of *nte*, the real trust value of an attacker is randomly assigned in the interval (0, δ]. However, collusive SSDF attackers will fake sensing data on the basis of maintaining their trust value in the interval [δ, 1]. Without loss of generality, we employ the averaged *nte* data of 100 cycles as the simulation results. As shown in Figure 5, the XDA scheme can reduce *nte* effectively. Without any guard measures, the *nte* curve with Baseline increases rapidly. By perfecting trust management after collusive SSDF attackers are detected, the *nte* curve with XDA increases smoothly.

Finally, we validate the performance of XDA in terms of attack success ratio when attackers launch collusive SSDF attackers with high trust value. This simulation is performed at two types of attack patterns including monopolizing vacant PU spectrums and disturbing data fusion. Without loss of generality, we employ the averaged attack success ratio data of 50 rounds of attack as the simulation results. At each round of attack, several cooperating SUs are selected randomly to perform a CSS action from honest SUs and collusive SSDF attackers.

**Figure 6:**
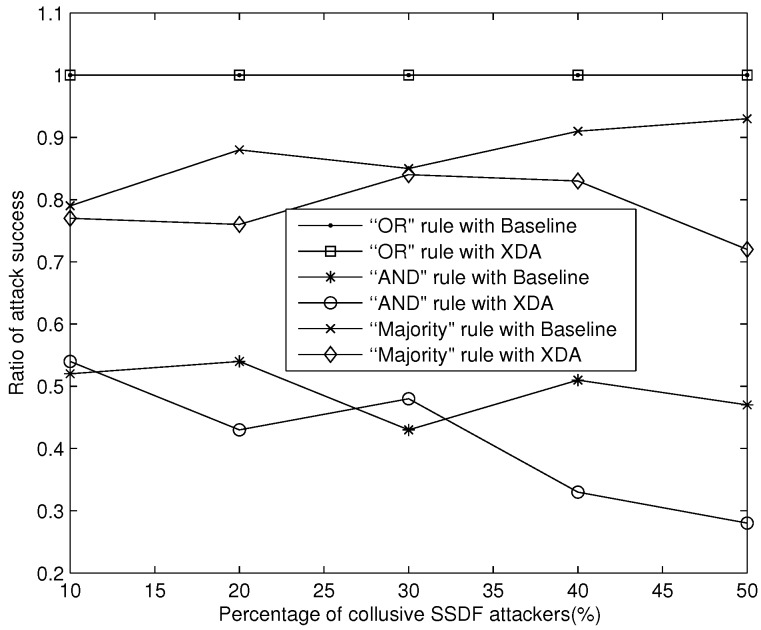
Suppressing MAC attack success ratio against monopolizing vacant PU spectrum.

As shown in Figure 6, the XDA scheme can suppress the attack success ratio better than Baseline under the “AND” and “Majority” rule when collusive SSDF attackers fake all “1” sensing data to monopolize vacant PU spectrums. In the “OR” rule, only one false “1” sensing data can mislead the final decision as “1”. Both of the schemes are difficult to defend against collusive SSDF attack. Therefore, collusive SSDF attackers can easily monopolize vacant PU spectrum in the “OR” rule, and thus gain exclusive access to the target licensed spectrum. In summary, to gain a reliable final decision against the threat of monopolizing vacant PU spectrums, the “OR” rule may not be a good choice.

**Figure 7:**
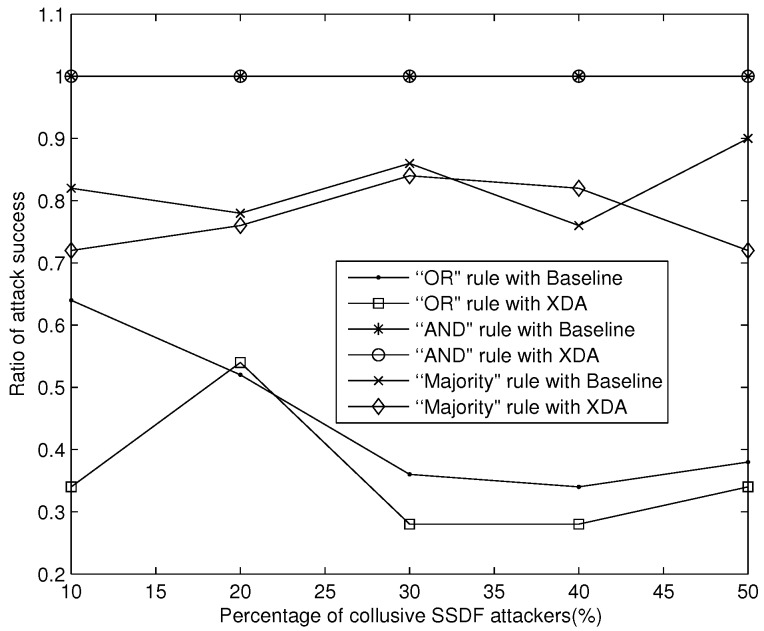
Suppressing MAC attack success ratio against disturbing data fusion.

We can also see in Figure 7 that the XDA scheme can suppress the attack success ratio better than Baseline under the “OR” and “Majority” rule when collusive SSDF attackers fake all “0” sensing data to disturb data fusion. In the “AND” rule, only one false “0” data can mislead the final decision as “0”. Both the two schemes are difficult to defend against collusive SSDF attack. Therefore, collusive SSDF attackers can easily disturb data fusion in the “AND” rule, and thus deceive honest SUs to interfere with PUs. In summary, to gain a reliable final decision against the threat of disturbing data fusion, the “AND” rule may be not a good choice with the threat of MAC attack.

## 5. Conclusions

In this paper, we analyzed the threats of collusive SSDF attack and proposed the XDA scheme to suppress such attacks. The XDA scheme was conducted in three successive stages: XOR distance calculation, collusive SSDF detection and perfect trust mechanism, in which XOR clustering analysis is introduced to design the XDA scheme due to the type of “0” and “1” historical sensing data. Simulation results show that our XDA scheme can enhance the accuracy of trust evaluation and suppress the collusive SSDF attack success ratio to some extent.

## Figures and Tables

**Table 1:** Description of the 0-1 database style.

Sensing Times	ID(Sensing Data)					
1	$SU_{1}{\left(d_{1}\right)}_{1}$	$SU_{2}{\left(d_{2}\right)}_{1}$	⋯	$SU_{i}{\left(d_{i}\right)}_{1}$	⋯	$SU_{n}{\left(d_{n}\right)}_{1}$
2	$SU_{1}{\left(d_{1}\right)}_{2}$	$SU_{2}{\left(d_{2}\right)}_{2}$	⋯	$SU_{i}{\left(d_{i}\right)}_{2}$	⋯	$SU_{n}{\left(d_{n}\right)}_{2}$
⋯	⋯	⋯	⋯	⋯	⋯	⋯
*k*	$SU_{1}{\left(d_{1}\right)}_{k}$	$SU_{2}{\left(d_{2}\right)}_{k}$	⋯	$SU_{i}{\left(d_{i}\right)}_{k}$	⋯	$SU_{n}{\left(d_{n}\right)}_{k}$

**Table 2:** Description of simulation elements.

Parameters	Description	Default
$N_{s}$	Number of SUs	60
$N_{p}$	Number of PUs	3
*cycle*	Number of cycle simulation	100
*round*	Rounds of attack	50
$p_{a}$	Percentage of attackers	10∼50%
δ	Threshold of trust value	0.8

## Abbreviations

The following abbreviations are used in this manuscript:

CSS	Cooperative Spectrum Sensing
SU	Secondary User
PU	Primary User
FC	Fusion Center
SSDF	Spectrum Sensing Data Falsification
XDA	XOR Distance Analysis
